# Grieving in virtual worlds: emotional processes and generational differences in avatar-based memorials on VRChat

**DOI:** 10.3389/fpsyg.2025.1669020

**Published:** 2025-12-10

**Authors:** Mengxi Fu, Hanyu Xiao, Haoyi Ruan, Yuran Lin, Xiangyu Dong

**Affiliations:** 1 Design and Performing Industries Department, Wales Institute of Science and Art, University of Wales Trinity Saint David, Swansea, United Kingdom; 2 Communication and Design Art College, Yunnan University of Finance and Economics (YNUFE), Kunming, China; 3 Innovation School of Greater Bay Area, Guangzhou Academy of Fine Arts, Guangzhou, China

**Keywords:** virtual mourning, grief, avatar, generational differences, digital death, emotion

## Abstract

**Introduction:**

Digital media and immersive technologies have introduced new modes of grieving, particularly within virtual reality (VR) environments where users inhabit emotionally meaningful avatars. While existing research focuses mostly on commemorating real-world deceased individuals, little is known about how people experience grief when virtual avatars—embodied extensions of identity—are lost, deleted, or symbolically “died.” This study addresses this gap by examining generational differences in how users interpret and respond to avatar loss within VR-based memorial ceremonies.

**Methods:**

Two avatar-based funerals were conducted on VRChat using qualitative research methods: a solemn ceremony for a retired World of Warcraft “Death Knight” and a playful sea-burial for a Sea of Thieves “Pirate.” Data were collected through participant observation and sixteen semi-structured interviews with users aged 18–65. Thematic analysis was conducted to identify key dimensions of emotional and cultural meaning.

**Results:**

Two overarching thematic dimensions emerged: (1) Generational Differences and Cultural Influence, and (2) Emotional Experience During Digital Mourning. Younger participants tended to approach avatar loss with humor, narrative play, and flexible identity experimentation, whereas older participants engaged with greater solemnity, ritual structure, and symbolic continuity in memorial practices.

**Discussion:**

Integrating grief theory with socio-technical perspectives, the study proposes the Hybrid Grief Model of Virtual Mourning (HGM-VM), conceptualizing digital grief as a dynamic interplay of emotional transition, oscillatory coping, and continuing bonds within virtual spaces. The findings show that virtual mourning does not diminish emotional authenticity but reconfigures it through immersive, participatory, and generationally shaped practices, offering theoretical and practical implications for designing inclusive and emotionally resonant virtual memorial environments.

## 1 Introduction

Historically, mourning rituals have been central to human societies, providing structured contexts for emotional expression, social cohesion, and collective memory. However, recent developments, particularly during and after the COVID-19 pandemic, have accelerated the transition toward virtual mourning practices (MacNeil et al., 2023). From online memorial pages and livestreamed funerals to virtual reality (VR) rituals, mourning practices have increasingly shifted from physical spaces into immersive digital environments (Pitsillides and Wallace, 2021). These practices—often classified under the broader category of thanatechnology—enable individuals to express grief, maintain symbolic bonds, and construct meaning through technologically mediated presence (Kohn et al., 2019; Kasket, 2019; Sumiala, 2021).

In this evolution, virtual reality platforms like VRChat are emerging as new spaces for mourning and remembrance. Unlike traditional funerals, virtual memorial ceremonies in VRChat allow participants to interact through personalized 3D avatars, fostering emotional engagement and communal support across vast physical distances (Kiros, 2023). The unique affordances of VR technology, including immersive experiences, personalized digital environments, and real-time interaction capabilities, have significantly influenced how grief is processed and expressed.

Within this context, virtual avatars operate as embodied interfaces carrying personal identity, emotion, and memory, often becoming objects of strong attachment. When these digital bodies are lost, deleted, or symbolically “killed,” users may experience genuine emotional distress and perform ritualized acts of remembrance (Banks and Bowman, 2014; Gibson and Talaie, 2018; Kiros, 2023). Despite the growing visibility of such practices, avatar loss remains an underexplored form of grief within digital death studies.

Most existing research focuses on commemorating real-world deceased individuals, analysing how bereaved users maintain online memorials or continue emotional bonds through social media (Brubaker et al., 2019; Mortazavi et al., 2021). Far less is known about how people experience the loss of virtual selves or avatars. This gap is important because avatar loss challenges conventional bereavement models; mourners may also be the creators or embodied operators of what has been lost.

Generational differences add further complexity. Younger users, shaped by digital culture, often view online commemoration as a natural and authentic mode of expression, while older users prefer private, solemn, and tangible rituals (King and Carter, 2022). Although prior work recognizes generational variation in digital mourning, few studies integrate grief theory to examine how these dynamics manifest in virtual rituals. Research seldom addresses how different generations navigate classical grief stages in VR settings (Akhther and Tetteh, 2021), nor does it consider the psychological and cultural implications of avatar disappearance. Little work combines psychological models with socio-technical analysis to explain how immersive media reshape emotional processes.

To address these gaps, this study investigates how different generations experience and interpret avatar loss within VR-based memorial environments. By connecting emotional expression, cultural meaning, and digital embodiment, it offers a deeper understanding of mourning practices in technologically mediated life. To achieve these objectives, two specific avatar-based memorial ceremonies were conducted on VRChat: one memorializing a World of Warcraft avatar known as the “Death Knight” and the other commemorating a Sea of Thieves avatar identified as the “Pirate.” These ceremonies serve as practical case studies, providing rich qualitative data through participant observation and in-depth interviews with participants across various age groups and cultural backgrounds.

The findings from this study will not only enhance theoretical understandings of grief and emotional processing in virtual contexts but also inform the design and implementation of future virtual memorial practices. As virtual and physical memorial practices continue to intersect and evolve, understanding emotional dynamics across generations will be increasingly critical for addressing diverse needs in memorial rituals.

## 2 Literature review

### 2.1 Theories of grief and digital adaptation

Traditional grief theories have long shaped psychological and sociocultural understandings of bereavement. Kübler-Ross’s (1969) five-stage model—denial, anger, bargaining, depression, and acceptance—remains one of the most influential frameworks (Bowlby, 1969; Kübler-Ross and Kessler, 2014; Sumiala, 2021). Subsequent models expanded its linear perspective. The Dual Process Model introduced an oscillation between loss-oriented coping (emotional confrontation) and restoration-oriented coping (adaptation and reconstruction), emphasizing fluidity rather than fixed stages (Stroebe et al., 2011; Bainbridge, 2014). Building on this development, the Continuing Bonds theory (Klass et al., 1996; Walter, 2015) redefined mourning as the ongoing maintenance of relational ties with the deceased rather than emotional detachment.

With the rise of digital communication, these models have been reinterpreted within technologically mediated environments (Irwin, 2015). For instance, recent studies have applied the Kübler-Ross model to analyse emotional responses to the deaths of fictional characters, revealing that users’ expressions of grief on social media mirror those observed in real-world bereavement (Şengün et al., 2023). Gender differences are also evident: men tend to employ humor to mask emotional reactions, whereas women express sadness and anger more directly (Sandra and Mutiaz, 2021). Online memorial pages, livestreamed funerals, and virtual reality (VR) rituals allow bereaved individuals to maintain symbolic interaction with the deceased and to reconstruct meaning through participatory engagement (Maddrell and Sidaway, 2010; Brubaker et al., 2019; Mortazavi et al., 2021). Digital mourning thus merges psychological processes with social performance, as platforms shape both affective expression and collective remembrance.

Whereas traditional grief theory focuses primarily on individual emotion, sociocultural perspectives highlight how mourning is mediated by collective practice and material media. Laqueur’s (2015) notion of the cultural work of the dead suggests that acts of commemoration always depend on ritual, narrative, and artifact (Laqueur, 2018). This study adopts that insight as a contextual framework: grief is approached here as a socially mediated and culturally embedded process, rather than as a purely individual response.

### 2.2 The disappearance of the avatar and digital embodiment

In emerging virtual-reality platforms such as Second Life and VRChat, avatars function as embodied interfaces that carry emotional, identity-related, and interactive significance. Research indicates that users often project aspects of their selfhood onto avatars and may develop strong emotional attachment; when avatars are harmed or disappear, users experience genuine psychological distress (Yee and Bailenson, 2007; Banks and Bowman, 2014; Dukes et al., 2021). This attachment transforms the avatar from a communicative tool into a vessel of memory and emotion. As an embodied extension of the self, the avatar enables users to perform commemorative gestures in immersive environments—speaking, bowing, or keeping vigil (Gibson and Talaie, 2018; Gibson, 2024). When an avatar is deleted or lost due to platform closure, technical failure, or intentional ritual, the resulting grief can be comparable to that caused by interpersonal loss (Kiros, 2023).

This phenomenon extends grief theory into new ontological territory where the boundary between subject and object becomes blurred and mourners simultaneously experience presence and absence (Beaunoyer and Guitton, 2021). Despite the increasing visibility of avatar loss in digital culture, its place within psychological and sociocultural frameworks of bereavement remains underexplored. Few empirical studies have examined how users interpret, express, or ritualize such experiences, nor how these responses vary across generations or cultural contexts.

### 2.3 Digital mourning and commemorative practices

Digital environments have expanded both the spaces and forms of remembrance (Moreman and Lewis, 2014). The concept of digital death refers to the ways online infrastructures mediate dying, mourning, and memorialization (Kasket, 2019; Öhman and Floridi, 2018, Biçer and Yıldırım, 2021). Virtual memorials reconstruct the traditional functions of cemeteries—remembrance, reflection, and communal connection—within digital domains (Giaxoglou et al., 2017). Visitors can walk through virtual spaces, light candles, or leave tributes. Such actions preserve the grammar of traditional ritual while introducing multisensory, networked experiences (Arnold et al., 2017; Ip, 2021). Interactive art projects such as Virtual Cemetery, along with VR installations that simulate funerary scenes, invite participants to confront death through immersive sensory engagement (Benski and Fisher, 2013; Roberts, 2004). Virtual memorials and livestreamed ceremonies render grief tangible, participatory, and unbound by geography (Haverinen, 2017; Sumiala, 2021; Sumiala and Jacobsen, 2024). Through these media, presence and absence intertwine—data, images, and interaction become new vessels of memory.

Empirical studies document how bereaved individuals use networked platforms to sustain continuing bonds with the deceased—posting messages, curating archives, or attending online vigils (Giaxoglou, 2014; Brubaker et al., 2019; Hasell, 2020). However, most of this literature focuses on commemorating real individuals and rarely addresses the disappearance of virtual identities. Certain cases, such as the memorial events following the closure of AltspaceVR, demonstrate how entire platforms may be anthropomorphized as virtual beings and become objects of mourning (Kentbye, 2023). This distinction highlights the multiple dimensions of “death” and “commemoration” in digital space. A central question thus arises: who—or what—is being remembered—the deceased person, the vanished digital persona, or the terminated platform itself?

Research on how users grieve for avatars—the immersive digital bodies that embody both personal and social meaning—remains scarce. This lack of attention underscores the importance of examining not only mourning through avatars but also mourning for avatars, whose disappearance can simultaneously disrupt emotional continuity and self-representation.

### 2.4 Generational differences in digital mourning

Generational background profoundly influences how individuals experience and express grief online (Sarkar and Banerji, 2024). Following sociological convention, this study distinguishes younger adults —combining Generation Z and Millennials—from older adults, encompassing Generation X and early Baby Boomers (Pew Research Center, 2019; Twenge, 2017). The two groups differ markedly in emotional expression, technological fluency, and ritual expectations.

Generational factors also shape how continuing bonds are maintained online. Studies show that younger cohorts are more likely to post directly to the deceased, maintain memorial pages, and regard these actions as extensions of relational interaction, whereas older cohorts are more inclined to close accounts, preserve archived content, or engage in private remembrance (King and Carter, 2022). Thus, while the continuing-bonds framework applies broadly, its concrete expression varies according to media literacy and cultural experience.

Younger participants, accustomed to networked communication, often perceive virtual memorials as authentic and emotionally resonant (King and Carter, 2022). They may express grief through creative play, humor, or aesthetic experimentation—forms that merge commemoration with social interaction. Older participants emphasize solemnity, privacy, and physical ritual, frequently viewing online or VR ceremonies as incomplete substitutes for embodied gatherings (Bell et al., 2015; Mou et al., 2023). These contrasts reveal that attitudes toward avatar loss and digital mourning are not purely psychological but also culturally generational, grounded in differing notions of authenticity and presence.

Although existing research confirms generational variation in digital emotional expression, few studies systematically integrate grief theory to analyse how these differences manifest within virtual commemorative rituals. In particular, there remains limited exploration of how younger and older cohorts experience the classic stages of grief differently in immersive virtual environments such as VR platforms (Akhther and Tetteh, 2021).

## 3 Methodology

### 3.1 Research design

This study employed a qualitative approach to explore emotional experiences and generational differences in virtual memorial practices, specifically focusing on virtual funerals held on the social VR platform VRChat. Qualitative methods were chosen to provide in-depth understanding and interpretative insights into participants’ subjective experiences of grief, memorialization, and digital identity.

Ethical approval was obtained from the University of Wales Trinity Saint David (UWTSD) Research Ethics Committee to ensure participants’ rights and confidentiality.

### 3.2 Research setting

The fieldwork was conducted virtually within VRChat, a popular, accessible, and interactive virtual reality platform, noted for its extensive user-generated content and diverse, predominantly younger user base. Two virtual memorial events were hosted in a researcher-designed digital environment, “Sherry’s Funeral House,” specifically created for commemorative rituals. Participants accessed the events through VR head-mounted displays (HMDs) or standard PC setups, with options to view livestreams and engage in interactive discussions.

#### 3.2.1 Create a digital funeral home, Sherry’s Funeral House on VR Chat

The practical component is essential to this research. A social-experimental artistic practice allows researchers to explore and understand specific phenomena based on their own experiences and perspectives. In this study, the researcher set up a digital funeral hall to hold a digital farewell ceremony for a user’s World of Warcraft game avatar. Participants could join the ceremony directly in VR Chat via VR HMD (like Quest 2) or a PC, and spectators could also watch the ceremony live.

#### 3.2.2 About Sherry’s funeral house

Sherry’s Funeral House is a digital farewell hall, an art performance research project on digital identity and memorialization. The researcher designed and uploaded the map to the VR Chat platform to host memorable digital farewell ceremonies, commemorating beloved avatars and digital identities in virtual worlds. The farewell ceremony is significant for the “deceased” and is therefore by invitation only. Invited guests need to prepare their attire and mood, read and sign an entry consent form and follow the host’s guidance to complete the ceremony.

Upon establishing a line of communication with participants, the researcher gathered data on the “deceased” avatar’s background and design, and formulated a plan for the ceremony. Utilizing the Unity 2019 4.3 game engine and Photoshop 2020, the research team built a custom funeral scene. Following this, the virtual farewell ceremony was conducted online, and the entire event was recorded and streamed with participants’ consent. Afterward, the researchers conducted semi-structured interviews with participants, combined with observations and reflections on the event.

### 3.3 Data collection and analysis

#### 3.3.1 Participants

Participants were recruited using convenience and snowball sampling methods via social VR forums (e.g., Reddit-VRChat) and direct in-platform recruitment. The study included a total of 16 participants, aged between 18 and 65 years, representing diverse cultural backgrounds primarily from Mainland China, the United States, the United Kingdom and Japan. Participants were required to have prior experience in VR. This recruitment approach was chosen because avatar-based mourning remains a niche and hard-to-reach phenomenon, making random sampling unfeasible. However, the method inherently introduces self-selection bias: individuals who are already emotionally expressive or comfortable discussing loss in virtual spaces may have been more inclined to volunteer. As a result, the sample may overrepresent participants with higher digital fluency or openness toward emotional engagement online. Besides, the sample remained small and unbalanced across regions, limiting cross-cultural generalization. These limitations are acknowledged and discussed in the final section.

Following established conventions in sociological and digital media research, this study divided participants into two generational cohorts to examine potential differences in emotional expression and technological adaptation. The boundary was set at 36 years old, distinguishing younger adults (18–35 years) from older adults (36–65 years).

This classification aligns with definitions proposed by the Pew Research Center (2019), which identifies Millennials (born 1981–1996) and Generation Z (born 1997 onward) as distinct from Generation X (born 1965–1980) and early Baby Boomers. Consequently, individuals aged 18–35 primarily represent digital natives—those who grew up with ubiquitous internet access and interactive media—whereas participants aged 36–65 represent digital immigrants, whose digital engagement developed later in life (Prensky, 2012; Helsper and Eynon, 2009; Twenge, 2017).

Beyond sociological classification, the 36-year threshold also corresponds to a developmental transition recognized in psychology. Erikson’s (1968) psychosocial model situates the mid-thirties as the shift from young adulthood (focused on intimacy and exploration) to middle adulthood (emphasizing stability and generativity). This age thus marks not only a technological divide but also a psychological and experiential boundary influencing communication, attachment, and emotional regulation. In the context of this research, such differentiation is particularly relevant to virtual mourning practices.

#### 3.3.2 Data collection

Data was collected through participant observation and semi-structured interviews.

##### 3.3.2.1 Participant observation

Researchers actively observed two virtual funerals—the “Death Knight” from *World of Warcraft* and the “Pirate” from *Sea of Thieves*. Observations focused on emotional expressions, interactions, ritual participation, and community dynamics.

##### 3.3.2.2 Semi-structured interviews

Sixteen semi-structured interviews were conducted through Discord, VRChat, Engage XR, VooV, or Zoom, based on participants’ preferences. Interviews averaged 40 min, were audio/video recorded, and focused on: Participants’ emotional experiences during virtual funerals; Perceptions of digital death and avatar attachment; and Generational differences in attitudes and behaviors. Interview questions included:

1. “How did you feel participating in a virtual funeral?”
2. “Could you describe memorable experiences involving your avatar?”
3. “How do you perceive the difference between virtual and traditional funerals?”
4. “What are your views on digital death and virtual memorials?”

Interviews were designed to encourage open-ended, phenomenological narratives from participants, promoting detailed and personal accounts of their virtual memorial experiences.

#### 3.3.3 Data analysis

Data analysis followed Braun and Clarke’s thematic analysis method (2006). This method was chosen for its flexibility and suitability for capturing the emotional processes and meaning-making embedded in participants’ narratives. All analysis was conducted manually to maintain close interpretive engagement with the data. The procedure was iterative and reflexive rather than strictly linear, cycling between stages to refine meanings and coherence.

**Step 1: Familiarizing with the Data**

All interview recordings were transcribed verbatim, including pauses, laughter, and tone shifts. Data familiarization involved repeated reading of transcripts, field notes, and related digital materials (e.g., chat logs, screenshots). Initial impressions, emotional cues, and recurrent ideas were annotated, treating transcription as an early interpretive act.

**Step 2: Generating Initial Codes**

Inductive, manual coding was conducted line-by-line. Codes were assigned to segments relating to emotional responses, symbolic expressions, and social interactions surrounding avatar loss and memorial practices. Both semantic and latent meanings were captured, with color-coding used to group similar concepts while retaining contextual detail. Equal attention was given to all data items to avoid anecdotal bias (Braun and Clarke, 2021).

**Step 3: Searching for Themes**

Codes were collated into broader candidate themes reflecting shared patterns across participants. Related codes—such as avatar attachment, ritual participation, and digital continuity—were organized into thematic clusters. Visual mapping and spreadsheets supported the identification of connections across emotional processes, identity transitions, and generational differences.

**Step 4: Reviewing Themes**

Themes were refined through iterative review. First, coded extracts were checked for internal coherence and reorganized where necessary. Second, themes were compared against the full dataset to ensure representativeness and conceptual clarity. Redundant or overlapping ideas were merged, and overlooked nuances were incorporated as the theme map was revised.

**Step 5: Defining and Naming Themes**

Each theme was clearly defined and delimited, with concise statements articulating its core meaning. Themes were situated within grief theory (Kübler-Ross and Kessler, 2014; Stroebe and Schut, 2010; Klass et al., 1996; Avis et al., 2021) to clarify their contribution to understanding avatar-related grief. Subthemes were added where needed, such as generational differences in emotional expression.

**Step 6: Producing the Report**

Themes were integrated into a coherent analytic narrative linking empirical observations with theoretical insights. Illustrative excerpts were selected to convey participants’ emotional experiences. Credibility was ensured through triangulation of interviews, participant observations, chat logs, and prolonged immersion in VRChat; transferability was supported through detailed contextual description.

#### 3.3.4 Ethical considerations

Due to the sensitive nature of death and grief, participants were provided informed consent and were informed of potential emotional risks. Procedures ensured anonymity and confidentiality of participants’ identities. Measures were in place for participants experiencing distress during events or interviews, including the right to withdraw at any stage.

#### 3.3.5 Reflexivity

The researcher maintained an explicit reflexive stance, recognizing personal cultural background, technological interests, and multicultural experiences influencing the interpretation and interactions with participants. Continuous reflexive practices were implemented to mitigate bias, including detailed self-reflection logs and supervisory consultations.

This methodological framework ensured the research findings authentically represented participants’ emotional experiences and accurately identified generational nuances in virtual memorial practices.

## 4 Results

### 4.1 Analytical framework

**FIGURE 1:**
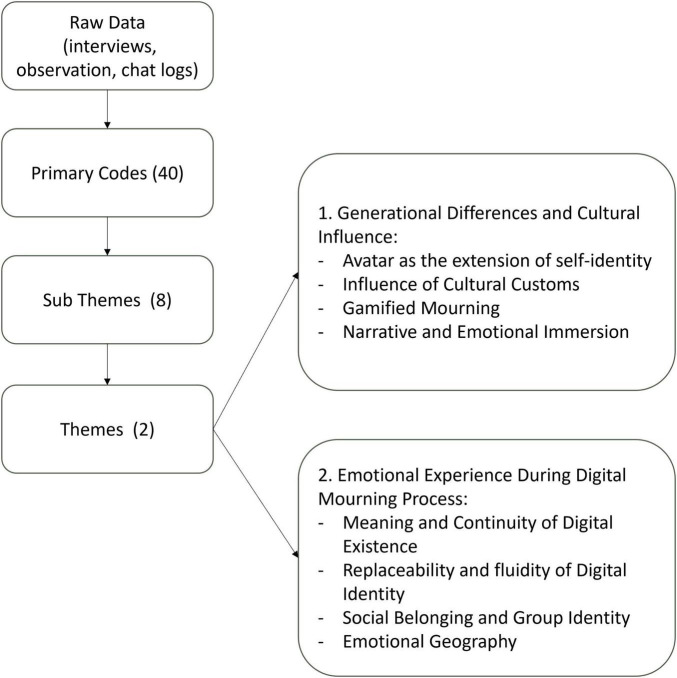
Thematical analysis process and findings.

Through thematic analysis of qualitative data collected from participant observations and semi-structured interviews conducted during avatar-based memorial events in VRChat, two overarching themes and eleven distinct sub-themes emerged. The first core theme, “Generational Differences and Cultural Influence,” comprises four subthemes:(1) Avatar as the extension of self-identity, (2) Influence of cultural customs, (3) Gamified mourning, and (4) Narrative and emotional immersion. Together, these subthemes illustrate how age, cultural background, and traditional practices significantly shape participants’ emotional responses and their acceptance of virtual memorial practices. The second core theme, “Emotional Experience During the Digital Mourning Process,” includes four subthemes: (1) Meaning and continuity of digital existence, (2) Replaceability and fluidity of digital identity, (3) Social belonging and group identity, and (4) Emotional geography. These subthemes highlight the diversity of emotional experiences expressed by participants and demonstrate how virtual environments facilitate unique forms of emotional connection, narrative participation, and interpretations of digital loss. These findings are summarized in Figure 1.

The analytical framework was informed by established grief theories. The five-stage model (Kübler-Ross and Kessler, 2014) was used to trace emotional transitions from denial, anger, bargaining, and depression to acceptance. The Dual Process Model (Stroebe and Schut, 2010) was applied to interpret the oscillation between loss-oriented coping (mourning the digital loss) and restoration-oriented coping (re-engaging with virtual communities). In addition, the Continuing Bonds Theory (Field, 2006) helped illuminate how digital memorials sustain symbolic and emotional bonds with deceased or retired avatars.

The study focuses on two representative cases: (1) PD’s memorial event, a commemorative gathering for a *World of Warcraft* character following the shutdown of its server; and (2) Xiang’s farewell ritual, a humorous sea-burial held in *Sea of Thieves* for a voluntarily retired avatar. Each case is analysed through three major dimensions—identity and thematic orientation, emotional trajectory, and generational perspective—and is subsequently synthesized into the proposed Hybrid Grief Model for Virtual Mourning (HGM-VM).

### 4.2 Event 1: the funeral of the death knight (World of Warcraft)

#### 4.2.1 Event process

To commemorate the permanent shutdown of his *World of Warcraft* account, PD commissioned the creation of a virtual church in VRChat—an elaborate architectural space inspired by gothic cathedrals, adorned with Death Knight motifs, and accompanied by PD’s own chosen soundtrack. The event was designed as both a memorial and a farewell ritual, inviting his former teammates to gather, perform blessings, and witness the symbolic departure of his avatar.

Prior to the ceremony, guests received detailed instructions and were asked to download VRChat to familiarize themselves with its navigation. Upon entering the virtual environment, they encountered an interactive entry consent form, which they clicked to proceed—a symbolic threshold between the everyday and the ritual space. Participants first assembled in the parlor, where snacks were displayed on tables to simulate social hospitality, before being guided into the main hall, a vast chamber featuring mirrors that allowed them to adjust their avatars’ appearance and composure before the ceremony began.

**FIGURE 2:**
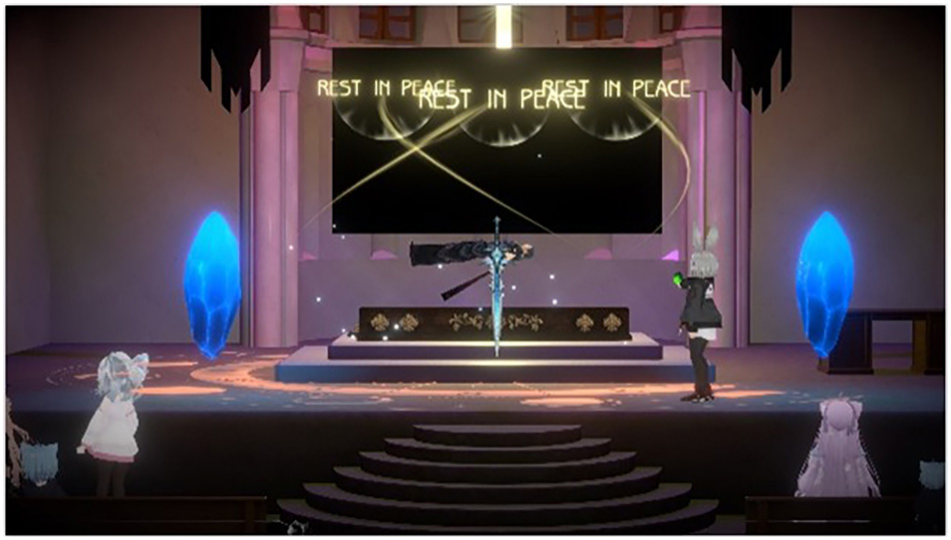
On-side event screenshot of the death knight’s funeral.

The memorial unfolded as a carefully staged sequence. The host, serving as master of ceremonies, invited attendees to stand before the altar while solemn music played. One by one, participants approached the platform during the blessing session, delivering personal tributes and farewell messages to the “deceased.” Each blessing triggered a transient visual effect—a shimmering light that gradually faded, marking the symbolic absorption of the prayer before the guest returned to their seat. The ritual culminated in the sending-off: a moment of “sacred light transmission” that enacted the avatar’s burial and transcendence to another world. Following the ceremony, guests gathered for group photographs and informal interviews, sharing reflections on their shared history within the game (Figure 2).

From the researcher’s observation, the emotional atmosphere evolved markedly over time. Initial laughter and casual conversation gave way to palpable nostalgia and grief as former teammates recounted shared battles and in-game achievements. When one participant quietly reached for a tissue to wipe away tears, the virtual illusion momentarily transformed into an authentic emotional experience—a collective enactment of mourning that blurred the line between digital performance and genuine loss.

#### 4.2.2 Theme identification: event 1

Event 1 illustrates the deep emotional investment that long-term players develop toward their avatars, treating them as meaningful extensions of the self. Several key themes emerged from the analysis (see Table 1 below for details).

**TABLE 1:** Thematic identification from PD’s memorial event.

Sub-themes	Description	Quote example
Avatar as the extension of self-identity	Participants reflected on how their digital personas embody real-life dispositions, values, and behavioral tendencies.	*“I’m the kind of person who, in real life, fights back when attacked—so I chose to be a fighter in the game.”* — GG
Influence of cultural customs	PD felt a conflict between maintaining the silent role of the deceased in the virtual coffin and the culturally informed expectation to reciprocate social interaction.	*“At that time, I was lying in the coffin. When the mourners performed certain actions or spoke to me, I quickly sent some emojis in response. I felt it would be impolite not to reply, yet as the deceased, I couldn’t speak or move, so I expressed myself only through emojis. It felt strangely fragmented.”* — PD
Meaning and continuity of digital existence	Participants linked digital persistence with ongoing symbolic existence, emphasizing memory, data, and collective recall.	*“I think a digital farewell is still a kind of memorial. As long as the data remains, it isn’t really death. A real death happens only when you no longer exist in someone else’s memory.”* — YK
Emotional geography	The significance of shared virtual environments was highlighted as essential to emotional connection and ritual authenticity.	“…*because World of Warcraft has shut down, they can’t hold such events inside the game anymore, so it becomes meaningful to recreate the scene in VR. It’s just like how I’d prefer us to do the interview on VRChat, because that’s where our shared experiences actually took place.”* — Fox

Taken together, these themes reveal how emotional attachment becomes transmitted and reinforced through ritual practice in virtual environments. Mourning, in this context, operates simultaneously as a form of emotional expression and a performative act mediated by digital affordances.

The tension PD described—between fulfilling social expectations and embodying the role of the silent deceased—highlights a hybrid ritual agency unique to virtual mourning: the deceased can “participate” without breaking ritual conventions, using minimal expressive cues such as emojis. This illustrates how continuing bonds are reconfigured in virtual form, allowing the bereaved and the “deceased avatar” to co-create meaning and emotional coherence during memorialization.

Furthermore, the theme of emotional geography underscores that place-making in virtual environments holds symbolic weight. The recreation of *World of Warcraft* spaces within VRChat is not merely nostalgic but functions as an attempt to restore the disrupted social–emotional landscape caused by the server shutdown. This supports the idea that digital memorials are not only commemorative acts but also spatial practices through which community memories are anchored and sustained.

### 4.3 Event 2: burial of the pirate (Sea of Thieves)

#### 4.3.1 Event process

The second memorial, titled “Burial of the Pirate,” took place within the maritime world of *Sea of Thieves* and served as a farewell rather than a death ritual. The event honored a long-standing pirate avatar whose user had decided to retire the character and migrate to another gaming community. Unlike the solemn tone of the *World of Warcraft* ceremony, this gathering reflected the pirate’s own wish for a sea burial infused with festivity and camaraderie, transforming loss into celebration.

The ceremony unfolded on a wide-open sea map at sunset, where several ships formed a circular flotilla to create a ritual arena. Participants arrived on deck dressed in their most elaborate pirate costumes, carrying lanterns and musical instruments. As the host announced the beginning of the farewell. Guests raised their tankards and cheered, flooding the chat with emojis and short messages of affection—digital gestures that blended humor with sincerity.

**FIGURE 3:**
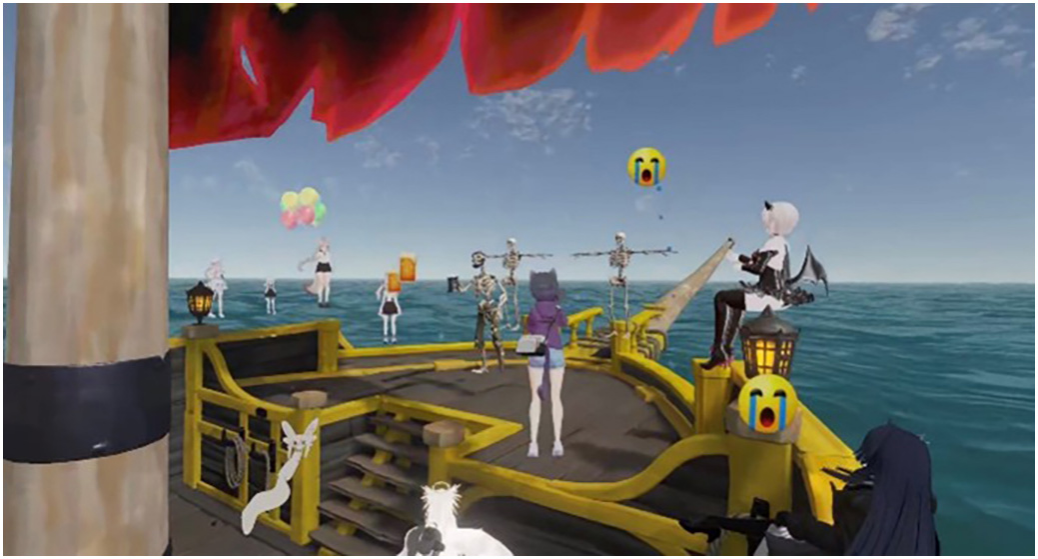
On-side event screenshot of the pirate’s funeral.

Despite its buoyant atmosphere, the event followed a ritual sequence similar to the previous funeral: a period of assembly, the reading of farewell messages, collective blessings, and finally, the symbolic burial at sea. The “body” of the pirate avatar was lowered into the waves on a makeshift raft, accompanied by luminous effects simulating dissolution into light and water. Throughout this process, laughter alternated with moments of silence, as friends shared recollections of past voyages and battles, narrating their adventures as a way of keeping the pirate’s memory alive (Figure 3).

From the researcher’s observation, the emotional tone was fluid—oscillating between jovial celebration and reflective melancholy. Younger participants embraced the playful, performative nature of the ritual, using virtual gestures and emojis to express affection, while older participants maintained a more restrained demeanor, quietly observing the spectacle. The fusion of festivity and farewell demonstrated how digital rituals can transform mourning into narrative continuity, turning the avatar’s departure into a communal story of transformation rather than an end.

#### 4.3.2 Theme identification: event 2

Xiang’s case involves younger participants and stands in sharp contrast to PD’s solemn memorial. This pirate-themed farewell ceremony reframed loss through creative play, humor, and collective interaction, transforming mourning into a socially performative and participatory event. See the Table 2. for the theme details.

**TABLE 2:** Thematic identification from Xiang’s farewell ceremony.

Themes	Description	Quote example
Gamified mourning	Younger participants demonstrated a greater openness toward digital commemoration, often approaching death-related topics through humor and playful reinterpretation.	“…*people of our generation, or those around our age, are beginning to transform how we talk about death. We tend to face such heavy topics with humor or other light-hearted approaches.”* — Ke
Replaceability and fluidity of digital identity	Participants emphasized the disposable or transformable nature of avatars, using them to explore or experiment with identity.	*“I believe that in VR, your avatar is inherently disposable — I could change my identity at any time.”* — FLOW
Social belonging and group identity	Emotional experiences were closely linked to social bonding within the game world.	“…*I feel quite happy when making new friends or when we all gather together. There are many kinds of groups in the game, and some people feel powerful or important because of the clubs they belong to.”* — Xiang
Narrative and emotional immersion	Participants stressed that a meaningful virtual death requires narrative significance and emotional depth, demonstrating how storytelling informs digital memorialization.	*“But for a character to be remembered, they need to have done something remarkable. There has to be a story about them — that’s what makes people remember and commemorate them.”* — Hong

These themes collectively illustrate a reversal of traditional mourning patterns. Instead of solemnity, the ceremony foregrounded humor, playfulness, and collaborative performance. Mourning became a creative act—a way for participants to reinterpret loss through shared storytelling, role-play, and social cohesion.

The playful tone did not diminish emotional authenticity; rather, it provided an alternative mode of processing grief. Humor functioned as a coping mechanism, allowing participants to approach the finality of avatar retirement in a manner aligned with generational attitudes toward death: flexible, ironic, and intertwined with everyday digital culture.

Overall, the case demonstrates that for younger users, virtual mourning is less about closure and more about creative continuity: sustaining bonds through play, narrative immersion, and group identity rather than traditional ritual solemnity.

### 4.4 Grief process

Among all interviewees, seven participants who were deeply involved in virtual reality memorial activities were selected for closer emotional-stage analysis. Their grief trajectories were examined using the Kübler-Ross model, as detailed in the table below. To illustrate fluctuations in emotional intensity throughout the mourning process, a five-point Likert-type continuum (1 = subdued emotion to 5 = highly expressive emotion) was used to provide a qualitative rating of each participant’s emotional expression (Table 3). These ratings were interpretive estimates based on narrative and behavioral cues—such as tone of voice, verbal emphasis, pauses, and embodied gestures—rather than self-reported scores. The aim was not quantitative measurement but the use of descriptive indicators to convey relative emotional intensity and clarify the progression across grief stages.

**TABLE 3:** Grief stage intensity scores (1–5 Likert scale).

Pseudonym	Denial	Anger	Bargaining	Depression	Acceptance
Xiang	3	1	3	3	5
PD	4	2	3	3	4
Yatan	2	1	3	4	5
Hong	3	1	2	3	4
Howie	3	2	2	3	4
Fox	2	2	2	4	5
Gan	2	1	3	2	4

As both participant and symbolic “deceased” in the Burial of the Pirate ceremony, Xiang’s emotional trajectory reflected a calm and pragmatic adaptation to loss. His initial reaction conveyed denial in a muted, reflective form—he acknowledged disappointment with the *Sea of Thieves* environment yet appeared emotionally detached, framing his withdrawal as a rational decision rather than an emotional rejection. Anger was nearly absent; he expressed no resentment toward the platform or other players, instead viewing the transition as a natural consequence of creative stagnation within the game. During bargaining, Xiang engaged in thoughtful conversations about how to preserve shared memories through screenshots and recordings, seeking symbolic continuity rather than reversal of loss. The depressive phase manifested subtly through nostalgic recollections of his crew’s adventures and a sense of melancholy over leaving a familiar digital community. Finally, Xiang reached full acceptance, demonstrating emotional maturity and openness to change—he embraced the farewell ritual as a meaningful closure and approached his migration to a new game world with optimism.

As both participant and the deceased, PD’s emotional journey intertwined humor and ritual control. His denial manifested through playful behavior—jokes and pranks masked the emotional impact of losing his *World of Warcraft* account. Beneath the humor, traces of anger surfaced as frustration toward the forced shutdown, sublimated through creativity. The bargaining phase appeared when PD transformed grief into action, designing the virtual church and orchestrating the memorial as a way to negotiate emotional meaning. During the ceremony, he entered depression, acknowledging the emotional gravity despite the festive tone; moments of silence revealed authentic sadness beneath performance. Finally, acceptance emerged through continuity—PD transitioned to other servers, integrating loss into his broader gaming life, exemplifying emotional adaptation and creative resilience.

Yatan’s emotional trajectory illustrates a gradual progression from cognitive hesitation to genuine emotional engagement. At first, he showed mild denial, surprised by how others were treating the virtual funeral with such solemnity, struggling to reconcile its gravity with the digital context. There was no explicit anger, though his nervousness hinted at discomfort with the emotional intensity of the event. During bargaining, he sought to make sense of the experience by contrasting virtual death with real-life mortality, reflecting on what counted as a “real” loss. As the ceremony deepened, Yatan entered a clear depressive phase—describing the atmosphere as “heavy” and emotionally charged, moved by teammates’ stories. By the end, he achieved acceptance, articulating a reflective understanding that virtual funerals can evoke authentic grief and foster empathy toward life beyond the screen.

Hong’s grief process began with denial marked by shock and contemplation—he struggled to grasp the significance of the event and hesitated to express overt emotion. Anger was absent, replaced by quiet confusion and internal questioning. His bargaining appeared intellectual rather than emotional; he analysed the meaning of virtual death as a mental negotiation about the boundary between the real and the digital. Entering depression, Hong described waves of sadness upon entering the virtual space, his mood fluctuating as memories surfaced. Ultimately, his acceptance took a reflective form: he rationalized the experience, acknowledging the symbolic power of virtual mourning while maintaining emotional distance, representing a measured reconciliation between thought and feeling.

Howie demonstrated restrained affect across all stages, reflecting an older participant’s reflective disposition. His denial was expressed as surprise and a certain detachment from the virtual environment—he found it difficult to emotionally engage with digital mourning. Anger was muted but discernible through mild frustration with the limitations of online rituals compared to physical funerals. Bargaining took the form of analytical reflection—he compared the sensory and moral dimensions of virtual versus real-world ceremonies. During depression, he expressed sadness when recalling traditional rituals that he felt provided deeper communal intimacy. By the stage of acceptance, Howie recognized the symbolic value of virtual mourning yet affirmed the irreplaceable authenticity of physical presence, achieving a balanced understanding of both.

Fox’s emotional pattern was dynamic and introspective. In the denial phase, curiosity and novelty dominated, masking any deeper sense of loss. Anger was not overt but emerged as subtle discomfort at the mixture of humor and mourning in others’ behavior. His bargaining phase was characterized by reflective questioning—whether virtual ceremonies could ever evoke the same sincerity as physical funerals. As eulogies unfolded, he entered depression, visibly moved and emotionally absorbed in the stories of shared adventures. Ultimately, acceptance arose through appreciation of the unique value of virtual funerals: he recognized their potential to reconnect dispersed communities and preserve social bonds, even while acknowledging their limitations in replicating embodied ritual.

Gan’s grief response was primarily cognitive rather than emotional. His initial denial appeared as detached curiosity, treating the event as novel and experimental. Anger was virtually absent; instead, he approached the experience pragmatically, focusing on its symbolic meaning. Bargaining surfaced through philosophical contemplation—he sought to reconcile the concept of virtual death with his understanding of human mortality. Depression appeared muted; he observed others’ emotions with quiet detachment, expressing a sense of resignation rather than sorrow. Finally, his acceptance reflected intellectual closure: he understood virtual death as a metaphorical process, accepting its philosophical implications more than its emotional reality.

## 5 Discussion

### 5.1 The virtual grieving process

The findings of this study indicate that mourning within virtual environments transcends the traditional linear trajectory proposed by Kübler-Ross (1969). Participants did not progress sequentially from denial to acceptance but oscillated between emotional states through mediated interactions—negotiating between presence, loss, and continuity. Based on the emotional analysis of multiple participants (see previous sections), and by integrating the Dual Process Model of bereavement (Stroebe and Schut, 2010) with the Continuing Bonds theory (Field, 2006), virtual mourning can be redefined as a hybrid system that combines emotional transition, adaptive oscillation, and enduring relational connection.

#### 5.1.1 Emotional transition: reconstructing the five-stage model

Within virtual mourning practices, the five stages are not discrete or terminal phases but fluid emotional transitions mediated by technology.

Shock and Denial often manifested as cognitive dissonance toward digital loss. Users questioned whether the disappearance of a virtual avatar equated to “death,” given the persistence of digital traces and the technical possibility of reanimation. This represents an early loss-oriented defense mechanism (Sandra and Mutiaz, 2021), intensified by the absence of culturally defined mourning scripts in virtual environments.

Anger, which in real-world bereavement typically targets specific agents or causes, was largely diffused in digital mourning. Instead, participants experienced frustration and helplessness in response to platform policies, data erasure, or server shutdowns, where no clear target for blame existed. The playful and participatory nature of virtual spaces often transformed anger into irony or collective empathy, neutralizing antagonistic emotions.

Bargaining appeared in restorative forms: participants sought to negotiate meaning between virtual and physical realms by preserving digital legacies, recreating memorial avatars, or performing ritualized log-ins. Such acts reflected oscillation between loss-oriented longing for memory preservation and restoration-oriented impulses toward reconstruction.

Depression arose from the strong emotional identification with avatars, leading to a perceived rupture in self-continuity. The disappearance of a virtual persona triggered existential questioning, disrupting coherence in identity and belonging. The lack of collective mourning frameworks within virtual platforms further deepened this sense of isolation.

Finally, acceptance emerged not as closure but as reconfiguration. Participants integrated loss into ongoing interaction by creating new avatars, curating digital relics, or transferring emotional investment to other communities—acknowledging impermanence while sustaining continuity. Acceptance thus functioned as adaptive integration rather than detachment.

Thus, these findings suggest that the five stages of grief in virtual mourning constitute an intertwined emotional flow shaped by platform affordances, generational attitudes, and cultural expectations. Emotional adaptation in virtual mourning is not a fixed sequence but a dynamic negotiation between technological mediation and human attachment.

#### 5.1.2 Oscillatory coping: the dual process of digital grief

The oscillatory nature of virtual grief aligns with the Dual Process Model of bereavement, where individuals alternate between confronting loss (loss-oriented coping) and reconstructing meaning or normalcy (restoration-oriented coping) (Avis et al., 2021). In virtual mourning, symbolic farewells express loss-oriented responses, while restoration-oriented coping emerges through identity reconstruction and renewed social belonging. Playful and participatory ritual elements diffuse emotional heaviness, transforming grief into creative engagement.

The contrast between PD and Xiang illustrates this dynamic. PD moved between mourning a lost avatar and reconnecting with guild members, balancing grief and restoration. Xiang, by contrast, displayed little anger or depression, showing pragmatic acceptance of digital impermanence. Shock and denial were brief; emotions alternated between nostalgia and renewal. This restoration-oriented mode emphasized creativity and gamified remembrance rather than linear emotional progression.

The two cases reveal complementary mourning patterns shaped by technology and generation. PD’s ritual embodied continuity, while Xiang’s reflected transformation and fluidity. Together, they illustrate a hybrid mourning process that integrates emotional attachment, technological affordance, and generational adaptation—both personal and collective, ritualistic yet playful. Virtual mourning thus operates as a cyclical negotiation of memory, identity, and social reintegration, underscoring the adaptive and fluid nature of digital grief.

#### 5.1.3 Continuing bonds: presence, memory, and digital relationality

From the perspective of Continuing Bonds theory, acceptance in virtual mourning signifies not closure but the continuation of a relationship. In virtual environments, avatars, memorial worlds, and preserved artifacts act as emotional anchors that sustain bonds beyond physical death. Users maintain presence and ritual continuity through ongoing interaction and shared commemorative narratives. As PD noted, *“Even though the server is gone, the videos, memories, and the funeral still exist—and that’s enough for me.”* His acceptance reflects transformation rather than detachment, as the avatar endures within the community’s collective memory.

This process supports Field’s view that maintaining symbolic bonds aids adaptation. In the digital afterlife, grief becomes dialogical: the living and the digitally deceased remain connected through interfaces, memories, and creative acts (Rovetta and Valentini, 2025). Participants often reconfigure emotional ties by integrating aspects of the deceased avatar into new works or narratives, granting a form of “virtual immortality.”

Ultimately, digital mourning reframes farewell as continuation—loss is integrated into evolving modes of presence, affirming that emotional persistence is central to mediated grief.

#### 5.1.4 The Hybrid Grief Model of Virtual Mourning (HGM-VM)

Integrating the above insights, this study proposes the Hybrid Grief Model of Virtual Mourning (HGM-VM), conceptualizing digital grief as a cyclical, multi-layered process composed of three interrelated dimensions:

1. Emotional Transition Layer – transforms the linear five-stage sequence into a fluid progression in which denial, bargaining, and acceptance coexist dynamically.
2. Oscillatory Coping Layer – incorporates the Dual Process Model to explain how individuals alternate between loss-oriented reflection and restoration-oriented reconstruction.
3. Continuing Bond Layer – emphasizes the maintenance of affective connections through digital artifacts, preserved avatars, and shared virtual spaces, redefining grief as ongoing existence rather than termination.

Within this framework, virtual mourning embodies both personal and collective dimensions, combining ritual solemnity with ludic creativity. Shaped by generational perception and technological mediation, it represents a hybrid emotional ecology in which grief is reconfigured as a process of relational transformation rather than closure. The virtual world thus becomes simultaneously a site of loss and a platform for continuity—extending the cultural function of mourning into the age of digital immortality.

### 5.2 Generational differences: emotion and ritual culture in virtual mourning

The Hybrid Grief Model of Virtual Mourning (HGM-VM) illustrates how individuals cope with loss and adaptation through emotional oscillation and continuing bonds. Yet mourning in virtual spaces is not only a psychological phenomenon but also a culturally embedded social practice. The ways users express, ritualize, and transform grief are shaped by generational experience, technological familiarity, and emotional socialization. In other words, while the hybrid model explains how grief unfolds, generational and cultural differences reveal why it manifests in different forms. This section shifts the focus from individual emotional mechanisms to sociocultural dimensions, exploring how distinct age groups construct unique “emotional grammars” and ritual preferences in virtual commemoration.

#### 5.2.1 Younger participants: gamified rituals and emotional fluidity

Younger participants tended to interpret avatar death as transformation rather than termination, viewing digital identities as fluid, adaptive, and replaceable. Their mourning practices combined humor, creativity, and collective participation—what can be described as gamified mourning.

This attitude was exemplified in Xiang’s *Sea of Thieves* funeral, which marked a symbolic closure rather than the end of existence: “*I still have many characters, but I wanted to give this one a proper ending so everyone could remember him.*”

For these participants, avatar loss was treated less as death and more as a stage of narrative completion. Their ceremonies often resembled festive celebrations—featuring jokes, music, and emojis—transforming grief into communal play. Rather than solemn remembrance, such rituals embodied light-hearted connection, reflecting a broader shift in emotional socialization within participatory digital culture (Walter, 2014). For digital natives, mourning became a shared creative performance, transforming loss into social cohesion.

As KE explained, the difference lies in how ritual seriousness is mediated by technology:

“*The sense of ritual usually needs physical elements—like the atmosphere of a memorial hall. In digital space, this can be simulated, but it’s like reading an e-book instead of a paper book. You focus differently. The digital medium often reduces the sense of solemnity, making commemoration feel more casual, even playful.*”

This perspective reveals a generational shift toward more positive and participatory engagement with virtual commemoration. While such practices may diminish the gravity of traditional mourning, they also foster a new emotional logic: grief becomes relational, shared, and reinterpreted rather than private and severing.

Interaction with the deceased’s avatar—through shared memories, symbolic gestures, or even humorous exchanges—extends relational presence across the digital–physical divide. Emojis, memes, and collective storytelling thus become tools for maintaining symbolic connections. Rather than static remembrance, these playful interactions constitute a living, dialogical bond, affirming that digital memorialization can sustain grief through continuity rather than closure.

#### 5.2.2 Older participants: ritual continuity and existential depth

In contrast, participants over 30 exhibited a markedly different emotional pattern. They tended to bind their virtual avatars closely to personal identity, viewing them not merely as game characters but as extensions of selfhood that embodied long-term relationships and lived experiences. PD’s *World of Warcraft* funeral for his Death Knight exemplified this. Having invested over a decade into the character, PD described the server shutdown as a genuine personal loss:

“*It wasn’t just the character disappearing—it felt like a part of me was gone. All the battle records, equipment, and friends’ messages vanished, as if I’d lost an old friend in real life.*”

Such reflections reveal that veteran players often form enduring attachments to their avatars, and the “death” of these digital selves evokes grief comparable to real-world bereavement. Older participants preferred formal, ritualized acts of mourning to assign symbolic meaning to the avatar’s death. During PD’s ceremony, guild members wore designated gear and stood in formation, following long-established traditions. This preference for solemn, structured commemoration contrasted sharply with younger players’ playful, celebratory approach.

Technological and emotional barriers further reinforced this orientation. Older users often faced accessibility or resonance challenges in digital environments (Fu et al., 2025) and therefore sought to reproduce the familiarity of traditional mourning—emphasizing structure, symbolism, and gravitas. For them, ritual enactment facilitated acceptance, reframing digital death not as disappearance but as transformation and continuity within collective memory.

These practices echo Laqueur’s (2018) concept of the work of the dead, wherein the living sustain the social vitality of the deceased through cultural labor. In digital contexts, older participants effectively transpose this labor into virtual worlds, reconstructing the solemnity and order of physical funerary traditions. Their emphasis on authenticity and ceremonial structure reflects both a desire for cultural continuity and a negotiation with the non-corporeal nature of digital mourning. The digital ritual thus becomes a site where embodied cultural memory encounters technological mediation.

#### 5.2.3 Intergenerational convergence of emotional expression

The generational gap in virtual mourning extends beyond technological proficiency—it reflects cultural and emotional orientations. Younger users’ humor and fluid identity performance align with the emotional socialization of the digital age, where feelings circulate through memes, posts, and participatory creation (Brubaker et al., 2013). In contrast, older users retain a pre-digital ethos of solemnity, reverence, and permanence. Having invested deeply in a single avatar, older players often experience grief akin to real-world loss, whereas younger users move on more readily to new experiences. As FOX explained:

“*If an important character dies, I wouldn’t throw a party. I’d build a monument or at least leave a corner for remembrance. In fact, I already have—I made a video.*”

His statement highlights older participants’ desire for permanence and authenticity in digital memorialization, contrasting with younger players’ emphasis on fluidity and creative renewal. These generational grammars illustrate a broader social transformation: digital natives turn loss into acts of co-presence and collective creativity, while digital immigrants preserve authenticity through ritual continuity. Both sustain continuing bonds, though through different means—one dynamic and participatory, the other structured and symbolic.

This convergence suggests that virtual mourning does not reject traditional mourning functions but reconfigures them through mediation. Bridging generational gaps requires memorial spaces that accommodate both informal and formal modes of expression. Younger users may prefer flexible, community-oriented environments encouraging spontaneous interaction, while older participants may seek structured, ceremonially resonant settings that evoke traditional mourning. Effective virtual memorials should balance solemnity and playfulness—offering structure for those seeking continuity and openness for those pursuing creative connection.

Ultimately, generational differences underscore the hybrid nature of digital death: simultaneously traditional and transformative, personal yet collective. Within the Hybrid Grief Model, these findings position generational culture as the social layer of digital grief—where individual emotion becomes shared symbolic practice, shaping how digital societies remember, mourn, and sustain the presence of the deceased.

## 6 Conclusion

This study investigated the emotional grieving processes and generational differences in avatar-based memorial ceremonies conducted within the virtual reality platform VRChat. Through empirical case studies of two virtual memorial events—commemorating the “Death Knight” from *World of Warcraft* and the “Pirate” from *Sea of Thieves*—the research collected extensive qualitative data using participant observations and in-depth interviews with individuals across diverse age groups and cultural backgrounds. By integrating thematic analysis with traditional grief frameworks such as Kübler-Ross’s five-stage model, this research explored how grieving processes manifest uniquely within virtual environments, and how generational differences shape emotional expression and ritual preferences.

### 6.1 Key findings

This study reveals that mourning in virtual environments constitutes a hybrid emotional and cultural process, shaped by technological mediation, generational culture, and evolving notions of identity and loss. Integrating the Dual Process Model and Continuing Bonds theory, the proposed Hybrid Grief Model of Virtual Mourning (HGM-VM) conceptualizes virtual grief as a dynamic system of emotional transition, adaptive coping, and enduring relational continuity.

Comparative analysis between the two research events demonstrates a complementary modes of mourning: ritual continuity and transformative play. Older participants exhibited deep identification with their avatars and sought meaning through structured, solemn rituals that reproduced traditional forms of commemoration. Younger participants, by contrast, reinterpreted loss through humor, creativity, and collective participation, transforming grief into shared acts of play and narrative renewal. Despite these generational differences, both groups maintained emotional bonds with the deceased avatars—one through symbolic permanence, the other through interactive re-engagement—revealing the persistence of relational continuity as the core of digital mourning.

Overall, the findings suggest that virtual mourning does not diminish the cultural or emotional significance of grief; rather, it reconfigures mourning as a mediated, participatory, and evolving practice. Digital environments extend the social and emotional life of the dead into networked spaces, illustrating that grief in the digital age is less an endpoint than an ongoing negotiation between memory, technology, and human attachment.

### 6.2 Theoretical contributions

This study contributes significantly to the existing body of emotional theories by critically assessing their applicability within virtual memorial contexts. It confirms that traditional grief models—particularly Kübler-Ross’s five-stage model and the Dual Process Model—retain substantial explanatory validity in virtual spaces, effectively capturing fundamental emotional stages and coping strategies observed during digital mourning (Şengün et al., 2023; Mortazavi et al., 2021). These models effectively capture the fundamental processes of denial, anger, adaptation, and meaning reconstruction that also manifest in avatar-based loss.

However, the findings also highlight critical theoretical limitations, particularly concerning the unique emotional complexities introduced by immersive virtual platforms and avatar-based identities. In response, this research calls for an expanded theoretical framework that integrates these digital-specific dimensions. The proposed Hybrid Grief Model of Virtual Mourning (HGM-VM) contributes to this theoretical development by reconceptualizing grief as a cyclical, hybrid process encompassing emotional oscillation, adaptive reconstruction, and continuing digital bonds. By situating grief within the intersection of psychology, technology, and culture, this model enriches existing emotional theories and extends their relevance to the posthuman condition of mourning in virtual environments.

### 6.3 Practical implications

Practically, the research provides valuable insights and concrete recommendations for designing virtual memorial spaces capable of accommodating generational differences effectively. To address diverse emotional needs across generations, virtual memorial design must carefully balance immersive realism, emotional authenticity, and cultural sensitivity.

Firstly, designers should emphasize creating immersive and customizable virtual environments that resonate emotionally across generational lines. This involves integrating culturally relevant symbols, traditional rituals, and familiar commemorative elements for older generations while simultaneously fostering interactive, informal, and playful emotional expressions valued by younger users.

Secondly, promoting emotional sharing through intuitive avatar-based interactions is crucial. Enhancing real-time communication, enabling personalized avatar representations, and creating community-oriented spaces can significantly strengthen emotional bonds and communal support, thereby enhancing collective mourning experiences for all participants.

Lastly, addressing technological usability and accessibility, particularly for older demographics less accustomed to digital interaction, is essential. User-friendly interfaces, straightforward navigation, and reliable technical support can mitigate technological barriers, thus encouraging broader generational participation and deeper emotional engagement.

In summary, virtual memorial practices that thoughtfully integrate immersive design, emotional authenticity, cultural appropriateness, and technological accessibility hold great potential to foster meaningful, inclusive, and emotionally resonant experiences across generational divides.

### 6.4 Limitations, transferability, and future research

While this research provides important qualitative insights into emotional grieving processes and generational dynamics within virtual memorials, it also acknowledges several limitations. The sample comprised sixteen participants, limiting claims to statistical generalizability. Such a small, culturally specific cohort—mostly from East Asia and Western Europe—reflects the interpretive rather than representative aim of qualitative inquiry. Mourning norms and digital practices may vary significantly across cultures, suggesting the need for broader cross-cultural exploration. The data’s platform specificity (VRChat) and the community-based recruitment approach may also have introduced self-selection bias, favoring participants already comfortable with emotional expression in immersive digital settings. Furthermore, the subjective interpretation inherent in thematic analysis may influence how emotional nuances are represented. Despite these constraints, the study demonstrates strong interpretive validity and transferability. Its purpose was not statistical generalization but to illuminate how individuals construct, express, and negotiate grief through avatars, providing contextually grounded insights into emotional adaptation in digital environments. The findings and emergent Hybrid Grief Model of Virtual Mourning (HGM-VM) present a conceptual foundation for understanding how loss, restoration, and continuing bonds co-exist in technologically mediated spaces.

Future research should aim to empirically test and refine this hybrid model. Large-scale quantitative studies could systematically examine variables such as avatar attachment, emotional oscillation, and generational preferences using validated psychological scales. Combining quantitative methods with qualitative insights would enable a more comprehensive understanding of emotional grieving processes and provide broader empirical validation for theoretical expansions suggested by this study. Additionally, cross-cultural comparative studies would enrich understanding of how cultural contexts further mediate emotional and generational variations in virtual mourning practices.

In conclusion, the findings from this study significantly enhance the theoretical understanding of grief and emotional processing within virtual environments, offering valuable insights for designing inclusive and emotionally resonant virtual memorial practices. As digital and physical commemorative practices continue to evolve and intertwine, ongoing research addressing these dynamics will be essential to effectively meet the diverse and evolving emotional needs of contemporary society.

## Data Availability

The raw data supporting the conclusions of this article will be made available by the authors, without undue reservation.
